# JOVR is Promoting Closer Academic and Scientific Ties between Europe and Iran

**Published:** 2011-10

**Authors:** Carl P. Herbort

**Affiliations:** 1 Inflammatory and Retinal Eye Diseases, Center for Ophthalmic Specialized Care, Lausanne, Switzerland; 2 University of Lausanne, Lausanne, Switzerland

In February 2010, Shahid Beheshti University of Medical Sciences (SBUMS) organised in Tehran a very high standard conference on ocular inflammation and uveitis. Several members of the executive committee of the Society for Ophthalmo-Immunoinfectiology in Europe (SOIE) took active part in that meeting and we thoroughly enjoyed the superb hospitality given to us by our Iranian colleagues. After 3 days of friendly contacts, stimulating exchanges and mutual convergence of opinions and interests, the idea emerged to work on one issue of Journal of Ophthalmic and Vision Research (JOVR) devoted to uveitis and inflammatory eye diseases. This partnership between the SOIE and SBUMS led to the project resulting today and publication of the present issue. Such collaborative work, building bridges between clinicians and researchers from different parts of the world, corresponds exactly to the philosophy of the SOIE, a society dedicated to teaching and promotion of knowledge in the field of uveitis and inflammatory eye diseases in Europe and beyond. Since its formal foundation in 2005 in Lausanne, Switzerland, SOIE has organised courses and meetings in Monte Carlo, Monaco in 2006, Krakow, Poland in 2007, Cappadocia, Turkey in 2008, the oasis of Tozeur, Tunisia in 2009, St. Petersburg, Russia in 2010, Bad Blumau, Austria in 2011 and Vilnius, Lithuania 2011. To expand its range of activities, a sister society the Society for ophthalmo-immunoinfectiology in Europe and the Middle-East (SOIEME) was established in 2011, in order to intensify contacts in that part of the world, an example of which is the collaboration that led to the present achievement.

This issue was planned to include and combine articles by Iranian authors on one side and from authors in connection with the SOIE on the other side. After a little more than one year of work and efforts, we succeeded to build an issue consisting of 14 peer-reviewed articles, equally divided between contributors from Iran and from the SOIE.

In this editorial, articles originating from the SOIE will be commented, while Professor Masoud Soheilian, the other guest-editor of this issue, will present Iranian contributions in his editorial.

In their original article, Papadia M and colleagues^1^ present a topic that, to the best of their knowledge, has never been dealt with at least in the last 30 years, namely the relation between myopia and inflammation. They describe on one hand myopic changes induced by inflammation including the myopic shift induced by sclero-choroidal inflammation that for example can occur in hyperacute Vogt-Koyanagi-Harada (VKH) disease or more rarely, by drugs such as acetazolamide or sulphonamides used in some cases of inflammation. On the other hand, they expose the most common clinical entities for which myopia can be a predisposing factor, such as multifocal choroiditis (MFC) and multiple evanescent white dot syndrome (MEWDS). Fragility of the choriocapillaris might be the common denominator for this association, as they hypothesize. The importance of indocyanine green angiography (ICGA) for these entities is put forward in this article.

As an illustration to this article, Bouchenaki and Herbort^2^ report a new approach for management of VKH disease. Progression of quasi 100% of VKH cases to sunset glow fundus even in the absence of clinically apparent disease assumes silent progression of choroidal disease that can only be detected by ICGA. This is what the authors indicate and they consequently decided to follow their VKH patients using ICGA and treat subclinical disease after resolution of inflammation. In patients treated early enough with ICGA monitoring, they avoided the evolution towards sunset glow fundus.

Three review articles on pediatric uveitis^3^, neurologic syndromes related with uveitis^4^, and biologic treatments for uveitis^5^ further widen the scope of this issue.

Pediatric uveitis is a topic that is not dealt with in a unanimous and consensual fashion, making it the subject of controversies. Ilknur Tugal-Tutkun, vice-president of the SOIE and one of the recognized uveitis specialists in Turkey, based her review on an extensive personal experience.^3^ Besides giving an exhaustive overview on childhood uveitis there are useful guidelines on management of pediatric cases indicating that corticosteroids should be replaced systematically by immunosuppressive treatment in case prolonged treatment is anticipated.

In their review on CNS diseases and uveitis, Allegri P, Rissotto R and colleagues^4^ provide an exhaustive account on neurologic diseases commonly associated with uveitis. Although the review is a collaborative work between a neurologist and ophthalmologists, the approach is from the neurologist’s point of view focusing on the neurologic side of these syndromes without neglecting the ophthalmological counterparts. This is probably interesting for ophthalmologists who are usually well informed on the ophthalmologic characteristics of these ocular/neurologic inflammatory concomitants but less so about their neurologic characteristics.

Piergiorgio Neri^5^, general secretary of the SOIE and one of the leading ocular immunologists in Italy contributes a short, albeit practical review on biologic treatments currently used in the management of non-infectious uveitis. Besides being a systematic review, it is based on long-standing experience, giving the clinician a present-day scope on these new and promising, sometimes sight-saving, therapeutic principles.

Finally, two interesting case reports showing the importance of ICGA in choroidopathies conclude the series of seven articles related to the SOIE. Papadia and Herbort^6^ present a case where central serous chorioretinopathy was mistaken for an inflammatory chorioretinopathy and treated with corticosteroids leading to a deleterious evolution. If ICGA signs had been taken into account, the diagnosis could have been made early without the consequences. Darugar and colleagues^7^ from the Pitié-Salpétrière hospital in Paris, housing one of the most important uveitis departments in Western Europe, present a case with typical clinical and ICGA findings of APMPPE. They stress the role of the choriocapillaris in the pathogenesis of APMPPE and remind us that a large range of differential diagnoses has to be considered before the condition can be termed idiopathic.

The collaborative effort between our two medical backgrounds resulted in the present issue of the JOVR that will hopefully interest ophthalmologists in both settings and will lead to closer contacts and better mutual appreciation and understanding.

We truly hope the reader will enjoy this special JOVR issue on uveitis and intraocular inflammation.

## References

[1] Herbort CP, Papadia M, Neri P (2011). Myopia and inflammation. J Ophthalmic Vis Res.

[2] Bouchenaki N, Herbort CP (2011). Indocyanine green angiography guided management of Vogt-Koyanagi-Harada disease. J Ophthalmic Vis Res.

[3] Tugal-Tutkun I (2011). Pediatric uveitis. J Ophthalmic Vis Res.

[4] Allegri P, Rissotto R, Herbort CP, Murialdo U (2011). CNS diseases and uveitis. J Ophthalmic Vis Res.

[5] Posarelli C, Arapi I, Figus M, Neri P (2011). Biologics for inflammatory eye diseases. J Ophthalmic Vis Res.

[6] Papadia M, Herbort CP (2011). Central serous chorioretinopathy mistaken for tuberculous choroiditis. J Ophthalmic Vis Res.

[7] Darugar A, Mathian A, LeHoang P, Bodaghi B (2011). Acute posterior multifocal placoid pigment epitheliopathy as the initial manifestation of sarcoidosis. J Ophthalmic Vis Res.

